# Transgender Women Experiencing Homelessness — National HIV Behavioral Surveillance Among Transgender Women, Seven Urban Areas, United States, 2019–2020

**DOI:** 10.15585/mmwr.su7301a5

**Published:** 2024-01-25

**Authors:** Ruthanne Marcus, Lindsay Trujillo, Evelyn Olansky, Susan Cha, Rebecca B. Hershow, Amy R. Baugher, Catlainn Sionean, Kathryn Lee, National HIV Behavioral Surveillance Among Transgender Women Study Group, National HIV Behavioral Surveillance Among Transgender Women Study Group, Narquis Barak, Kathleen A. Brady, Sarah Braunstein, Jasmine Davis, Sara Glick, Andrea Harrington, Jasmine Lopez, Yingbo Ma, Aleks Martin, Genetha Mustaafaa, Tanner Nassau, Gia Olaes, Jennifer Reuer, Alexis Rivera, William T. Robinson, Ekow Kwa Sey, Sofia Sicro, Brittany Taylor, Dillon Trujillo, Erin Wilson, Pascale Wortley

**Affiliations:** ^1^Division of HIV Prevention, National Center for HIV, Viral Hepatitis, STD, and TB Prevention, CDC, Atlanta, Georgia; ^2^Social & Scientific Systems, Inc., Silver Spring, Maryland; CrescentCare; Philadelphia Department of Public Health; New York City Department of Health and Mental Hygiene; CrescentCare; University of Washington, School of Medicine, Division of Allergy and Infectious Diseases, Public Health – Seattle & King County, HIV/STD Program; Philadelphia Department of Public Health; New York City Department of Health and Mental Hygiene; Los Angeles County Department of Public Health; Public Health – Seattle & King County, HIV/STD Program; Georgia Department of Public Health; Philadelphia Department of Public Health; Los Angeles County Department of Public Health; Washington State Department of Health; New York City Department of Health and Mental Hygiene; Louisiana State University Health Science Center in New Orleans – School of Public Health, Louisiana Office of Public Health STD/HIV/Hepatitis Program; Los Angeles County Department of Public Health; San Francisco Department of Public Health; Georgia Department of Public Health; San Francisco Department of Public Health; San Francisco Department of Public Health; Georgia Department of Public Health.

## Abstract

Transgender women experience high prevalence of homelessness, which can affect their likelihood of acquiring HIV infection and can lead to poor medical outcomes. CDC analyzed data from the National HIV Behavioral Surveillance Among Transgender Women to identify whether personal characteristics and social factors affecting transgender women were associated with duration of homelessness during the past 12 months. Longer duration and chronic homelessness might indicate greater unmet needs, which increases their likelihood for acquiring HIV infection. Ordinal logistic regression was conducted to calculate adjusted prevalence odds ratios and 95% CIs for transgender women from seven urban areas in the United States experiencing homelessness 30–365 nights, 1–29 nights, and zero nights during the past 12 months. Among 1,566 transgender women, 9% reported 1–29 nights homeless and 31% reported 30–365 nights homeless during the past 12 months. Among participants who reported physical intimate partner violence or forced sex, 50% and 47%, respectively, reported experiencing 30–365 nights homeless. Furthermore, 55% who had been evicted or denied housing because of their gender identity and 58% who had been incarcerated during the past year experienced 30–365 nights homeless. The odds of transgender women experiencing longer duration of homelessness was associated with being younger and having a disability; higher psychological distress scores were associated with longer duration of homelessness. Analysis of social determinants of health found transgender women experiencing longer homelessness to be less educated, living below the Federal poverty level, and having lower social support. Therefore, focusing on HIV prevention and interventions addressing housing instability to reduce the duration of homelessness among transgender women is important. Further, integrating housing services with behavioral health services and clinical care, specifically designed for transgender women, could reduce HIV acquisition risk and improve HIV infection outcomes.

## Introduction

Persons experiencing homelessness have increased risk for acquiring HIV infection and subsequent poor HIV outcomes (*1*). Transgender women account for <1% of the U.S. population (*2*), yet among transgender women, HIV prevalence rates up to 42% have been reported (*3*,*4*). Moreover, 39% of transgender women participating in the National HIV Behavioral Surveillance Among Transgender Women (NHBS-Trans) during 2019–2020 reported experiencing homelessness during the past 12 months (*3*,*5*). Housing instability, including homelessness, among transgender persons is often associated with poor medical outcomes (e.g., HIV and other viral infections), adverse mental health outcomes (*6*,*7*), psychological stressors (*8*), and lack of social support (*8*). A qualitative study of transgender persons found financial insecurity and interpersonal rejection by family and friends to be key factors associated with housing instability (*8*). These stressors often resulted in psychological strain and subsequent drug and sexual behaviors that increase the risk for HIV acquisition, including exchanging sex for money or drugs. Laws that discriminate against (*9*) and marginalize (*10*) transgender women further affect housing status and health outcomes. These policies are often fueled by societal transphobia (*11*,*12*). The duration of homelessness is associated with factors (e.g., substance use) that increase HIV risk among adults experiencing homelessness (*13*).

Housing instability can be dynamic, and definitions of homelessness vary, often related to duration of instability such as short-term or episodic (e.g., couch surfing, evictions, and frequent moves) versus longer-term or chronic homelessness (*14*). Although previous studies provided important information on factors associated with housing instability among transgender women, they involved limited samples in one location or specific subpopulations and did not assess associations between duration of homelessness during the past 12 months and the personal characteristics (e.g., age, race and ethnicity, HIV status, disability, and psychological distress) or social factors (e.g., education, insurance status, poverty level, experiences of abuse, eviction, being denied housing access, perceived social support, incarceration, and exchange sex) experienced by transgender women (*7*,*15*). The focus of this report is to identify specific personal characteristics and social factors associated with duration of homelessness defined as the number of nights spent homeless during the past 12 months among transgender women from seven urban areas in the United States. These findings can be used by housing services and health care providers to guide tailored HIV prevention and housing services for transgender women.

## Methods

### Data Source

This report analyzes survey data from the National HIV Behavioral Surveillance Among Transgender Women (NHBS-Trans) conducted by CDC during June 2019–February 2020 to assess behavioral risks, prevention usage, and HIV prevalence. Eligible participants completed an interviewer-administered questionnaire and were offered HIV testing. Additional information about NHBS-Trans eligibility criteria, data collection, and biologic testing is available in the overview and methodology report of this supplement (*16*). The NHBS-Trans protocol questionnaire and documentation are available at https://www.cdc.gov/hiv/statistics/systems/nhbs/methods-questionnaires.html#trans.

Applicable local institutional review boards in each participating project area approved NHBS-Trans activities. The NHBS-Trans sample included 1,608 transgender women in seven urban areas in the United States (Atlanta, Georgia; Los Angeles, California; New Orleans, Louisiana; New York City, New York; Philadelphia, Pennsylvania; San Francisco, California; and Seattle, Washington) recruited using respondent-driven sampling. This analysis is restricted to 1,566 participants with no missing information on homelessness during the past 12 months. This activity was reviewed by CDC, deemed not research, and was conducted consistent with applicable federal law and CDC policy.*

### Measures

Homelessness was defined as living on the street, in a shelter, in a single room occupancy hotel, or in a car during the past 12 months; participants who reported experiencing homelessness during the past 12 months were asked the number of nights they were homeless (Table 1). Participants were categorized as homeless 1–29 nights or 30–365 nights, or not homeless during the past 12 months; the number of nights did not need to be consecutive. These cut-offs were established to examine short-term or episodic homelessness and longer-term or chronic homelessness (*14*). Participants were asked about their personal characteristics and social factors.

**TABLE 1:** Variables, survey questions, measures, and analytic codings for personal and social variables among transgender women experiencing homelessness — National HIV Behavioral Surveillance Among Transgender Women, seven urban areas,* United States, 2019–2020

Variable	Question or measure	Analytic coding
**Personal characteristic**		
Age at interview, yrs	What is your date of birth?	18–24, 25–29, 30–39, 40–49, or ≥50
Race and ethnicity^†^	Do you consider yourself to be of Hispanic, Latino/a, or Spanish origin? Which racial group or groups do you consider yourself to be in? You may choose more than one option.	American Indian or Alaska Native, Asian, Black or African American, Hispanic or Latina, Native Hawaiian or other Pacific Islander, White, or multiple races or ethnicities
Disability status^§^	Are you deaf or do you have serious difficulty hearing? Are you blind or have serious difficulty seeing, even when wearing glasses? Because of a physical, mental, or emotional condition, do you have serious difficulty concentrating, remembering, or making decisions? Do you have serious difficulty walking or climbing stairs? Do you have difficulty dressing or bathing? Because of a physical, mental, or emotional condition, do you have difficulty doing errands alone, such as visiting a doctor’s office or shopping?	Yes to at least one of these six questions or no to all questions
Psychological distress^¶^	About how often during the past 30 days did you feel nervous? During the past 30 days, about how often did you feel hopeless? During the past 30 days, about how often did you feel restless or fidgety? How often did you feel so sad or depressed that nothing could cheer you up? During the past 30 days, about how often did you feel that everything was an effort? During the past 30 days, about how often did you feel down on yourself, no good, or worthless?	For respondents with nonmissing values on all six items, responses to all items were summed to create a psychological distress score (range = 0–24). Respondents with missing values for any of the six items were set to missing.
HIV status**	NHBS biologic HIV test result	Positive or negative
**Social factor**		
Education	What is the highest level of education you completed?	<High school, high school diploma or equivalent, some college or technical degree, college degree or more
Social support^††^	MSPSS — social support scale	Mean social support score (SD). Social support was calculated from 12 questions from three subscales of family support, friend support, and significant other support. Each item of the 12 questions had a five-point response option ranging from “strongly agree” (5) to “strongly disagree” (1).
Housing discrimination	In the past 12 months, have you been denied housing or been evicted because you are transgender or gender nonconforming?	Yes or no
Incarceration	During the past 12 months, that is, since [interview month] of last year, have you been held in a detention center, jail, or prison for more than 24 hours? Have you ever been held in a detention center, jail, or prison for more than 24 hours?	Incarcerated in the past 12 months; incarcerated, but not in the past 12 months; or never incarcerated
**Outcome variable**		
Homelessness	In the past 12 months, that is, since [interview month] of last year, have you been homeless at any time? By homeless, I mean you were living on the street, in a shelter, in a single room occupancy hotel, or in a car. If yes, about how many total nights were you homeless?	1–29, 30–365, or 0

**Abbreviations:** MSPSS = Multidimensional Scale of Perceived Social Support; NHBS = National HIV Behavioral Surveillance.

* Atlanta, GA; Los Angeles, CA; New Orleans, LA; New York City, NY; Philadelphia, PA; San Francisco, CA; and Seattle, WA.

^†^ Persons of Hispanic or Latina (Hispanic) origin might be of any race but are categorized as Hispanic; all racial groups are non-Hispanic.

^§^ Serious difficulty hearing, seeing, doing cognitive tasks, walking or climbing stairs, dressing or bathing, or doing errands alone. Adjusted for age. Based on U.S. Department of Health and Human Services disability data standard (https://aspe.hhs.gov/reports/hhs-implementation-guidance-data-collection-standards-race-ethnicity-sex-primary-language-disability-0).

^¶^ **Source:** Kessler RC, Barker PR, Colpe LJ, et al. Screening for serious mental illness in the general population. Arch Gen Psychiatry 2003;60:184–9.

** Determined based on NHBS rapid HIV test results.

^††^ **Source:** Zimet GD, Powell SS, Farley GK, Werkman S, Berkoff KA. Psychometric characteristics of the Multidimensional Scale of Perceived Social Support. J Pers Assess 1990;55:610–7.

Personal characteristics included demographics (age and race and ethnicity) and health status (HIV status based on National HIV Behavioral Surveillance HIV test result, disability status, and psychological distress). (Persons of Hispanic or Latina [Hispanic] origin might be of any race but are categorized as Hispanic; all racial groups are non-Hispanic.) Disability status was measured using the U.S. Department of Health and Human Services data standard for disability status (*17*). Psychological distress was measured with the Kessler Psychological Distress Scale (Kessler-6) (*18*). The Kessler-6 is a screening tool used to assess the prevalence of serious mental illness during the past 30 days, as defined by meeting criteria in the *Diagnostic and Statistical Manual IV*. Each question used a five-item Likert scale (4 = all of the time, 3 = most of the time, 2 = some of the time, 1 = a little of the time, and 0 = none of the time); responses were summed to an overall psychological distress score (range = 0–24).

Social factors were defined as education, health insurance status, and poverty level. Experience of abuse included experience of physical intimate partner violence, forced sex, or physical violence or harassment because of gender identity or presentation during the past 12 months. Social support was collected using the Multidimensional Scale of Perceived Social Support (MSPSS) (*19*), a 12-item scale used to measure social support of family, friends, and other special persons in the women’s lives. The scale uses a five-item Likert scale (5 = strongly agree to 1 = strongly disagree) for each question. Question scores were summed and averaged to calculate the overall social support score and SD. The MSPSS has demonstrated good internal validity among transgender women (*20*).

Additional social factors included whether the participant had been denied housing or been evicted during the past 12 months because they were transgender or gender nonconforming, had a history of incarceration, and had a history of exchanging sex for money or drugs (i.e., sex work). Housing discrimination was defined as being denied housing or being evicted during the past 12 months because they are transgender or gender nonconforming. Definitions of demographics and social determinants of health are available in the overview and methodology report in this supplement (*16*).

### Analysis

Univariate distribution of nights of homelessness was examined to determine appropriate cut-offs to classify short-term or episodic (1–29 nights) and longer-term or chronic homelessness (30–365 nights). Descriptive statistics of personal characteristics and social factors were conducted by the three-level outcome variable of duration of homelessness. The association between duration of homelessness and personal characteristics and social factors was evaluated through ordinal logistic regression analysis by using a proportional odds model. This approach was taken to evaluate the associations based on the ordinal outcome variable of nights of homelessness, on the basis of the assumption that these associations are homogeneous. The assumption of proportionality of the odds of the outcome was evaluated using the proportional odds score test, which tests the null hypothesis of no difference between the coefficients associated with the levels of duration of homelessness for transgender women. This method generated adjusted prevalence odds ratios and 95% CIs; models were adjusted for city of residence and network size and clustered on recruitment chain. Results were considered statistically significant if the 95% CI range did not overlap with the null (null = 1). Cronbach’s alpha was calculated to assess internal consistency of the MSPSS. Analyses were conducted using SAS software (version 9.4; SAS Institute).

## Results

Of the 1,608 NHBS participants, 42 were excluded from the analysis due to missing data on homelessness. Among these, 1,566 transgender women, 936 (60%) had not experienced homelessness during the past 12 months, 140 (9%) were homeless 1–29 nights, and 490 (31%) were homeless 30–365 nights (Table 2). Among those who were homeless 1–29 nights, the median number of nights homeless was seven, and the median number of nights homeless among those who were homeless 30–365 nights was 180. Experiencing 30–365 nights of homelessness was more prevalent among transgender women who were younger, had a disability, and were living at or below the Federal poverty level. Transgender women who had experienced any form of abuse (physical intimate partner violence: 49.8%; forced sex: 47.0%; physical violence: 42.9%) experienced 30–365 nights homeless. Transgender women who reported being evicted or denied housing because they are transgender or gender nonconforming (55.3%) experienced 30–365 nights homeless. Transgender women who had been incarcerated during the past 12 months (58.4%) and who had exchanged sex for money or drugs (43.6%) experienced 30–365 nights homeless.

**TABLE 2:** Number and percentage of transgender women experiencing homelessness,* by duration of homelessness during the past 12 months and selected characteristics — National HIV Behavioral Surveillance Among Transgender Women, seven urban areas,^†^ United States, 2019–2020^§^

Characteristic	Duration of homelessness, nights			Total no. (row)
	30–365	1–29	0	
	No. (row %)	No. (row %)	No. (row %)	
**Overall**	**490 (31.3)**	**140 (8.9)**	**936 (59.8)**	**1,566**
No. of nights homeless, median (IQR)	180.0 (60.0–365.0)	7.0 (4.0–14.0)	0.0 (—)	
**Demographic**				
Age group, yrs				
18–24	82 (43.6)	22 (11.7)	84 (44.7)	**188**
25–29	102 (33.9)	36 (12.0)	163 (54.2)	**301**
30–39	153 (33.6)	44 (9.6)	259 (56.8)	**456**
40–49	80 (27.1)	23 (7.8)	192 (65.1)	**295**
≥50	73 (22.4)	15 (4.6)	238 (73.0)	**326**
Race and ethnicity^¶^				
American Indian or Alaska Native	6 (42.9)	0 (0.0)	8 (57.1)	**14**
Asian	6 (20.7)	3 (10.3)	20 (69.0)	**29**
Black or African American	187 (33.5)	51 (9.1)	321 (57.4)	**559**
Native Hawaiian or other Pacific Islander	7 (16.7)	3 (7.1)	32 (76.2)	**42**
White	64 (36.6)	13 (7.4)	98 (56.0)	**175**
Multiple races	47 (40.5)	9 (7.8)	60 (51.7)	**116**
Hispanic or Latina	171 (27.2)	61 (9.7)	396 (63.1)	**628**
**Health status**				
NHBS HIV test result**				
Positive	213 (33.1)	57 (8.9)	373 (58.0)	**643**
Negative	262 (29.9)	82 (9.4)	532 (60.7)	**876**
Disability status^††^				
Has a disability	328 (39.6)	72 (8.7)	428 (51.7)	**828**
No disability	158 (21.6)	67 (9.1)	506 (69.2)	**731**
**Mental health**				
Psychological distress^§§^				
Score (mean [SD])	10.62 (5.59)	10.54 (5.44)	8.01 (5.11)	**9.05 (5.44)**
**Social factors**				
Education				
<High school	110 (32.8)	33 (9.9)	192 (57.3)	**335**
High school or equivalent	200 (34.1)	61 (10.4)	326 (55.5)	**587**
Some college or technical degree	145 (31.0)	33 (7.1)	290 (62.0)	**468**
College degree or more	34 (19.5)	12 (6.9)	128 (73.6)	**174**
Health insurance status				
Uninsured	88 (33.1)	36 (13.5)	142 (53.4)	**266**
Insured	401 (30.9)	104 (8.0)	794 (61.1)	**1299**
Poverty level^¶¶^				
At or below Federal poverty level	374 (38.1)	101 (10.3)	506 (51.6)	**981**
Above Federal poverty level	110 (19.3)	38 (6.7)	423 (74.1)	**571**
**Experience of abuse past 12 months**				
Physical intimate partner violence***				
Yes	119 (49.8)	31 (13.0)	89 (37.2)	**239**
No	370 (27.9)	109 (8.2)	846 (63.8)	**1,325**
Forced sex^†††^				
Yes	109 (47.0)	29 (12.5)	94 (40.5)	**232**
No	378 (28.4)	110 (8.3)	841 (63.3)	**1,329**
Physical violence^§§§^				
Yes	179 (42.9)	61 (14.6)	177 (42.4)	**417**
No	310 (27.0)	79 (6.9)	758 (66.1)	**1,147**
Social support scale^¶¶¶^				
Score (mean [SD])	3.47 (0.90)	3.70 (0.79)	3.85 (0.82)	**3.72 (0.86)**
**Evicted or denied housing because they are transgender or gender nonconforming past 12 mos**				
Yes	121 (55.3)	30 (13.7)	68 (31.1)	**219**
No	364 (27.2)	109 (8.1)	867 (64.7)	**1,340**
Incarceration****				
Never incarcerated	147 (22.5)	47 (7.2)	459 (70.3)	**653**
Incarcerated, not in past 12 months	186 (29.0)	57 (8.9)	398 (62.1)	**641**
Incarcerated in past 12 months	157 (58.4)	36 (13.4)	76 (28.3)	**269**
**Exchange sex for money or drugs^††††^**				
Yes	235 (43.6)	54 (10.0)	250 (46.4)	**539**
No	254 (24.8)	86 (8.4)	686 (66.9)	**1,026**

**Abbreviations:** IQR = interquartile range; NHBS = National HIV Behavioral Surveillance.

* N = 1,566 participants with no missing information on homelessness during the past 12 months.

^†^ Atlanta, GA; Los Angeles, CA; New Orleans, LA; New York City, NY; Philadelphia, PA; San Francisco, CA; and Seattle, WA.

^§^ Numbers might not add to total because of missing data. Percentages might not add to 100 because of rounding.

^¶^ Persons of Hispanic or Latina (Hispanic) origin might be of any race but are categorized as Hispanic; all racial groups are non-Hispanic.

** Participants with a reactive rapid NHBS HIV test result supported by a second rapid test or supplemental laboratory-based testing.

^††^ Serious difficulty hearing, seeing, doing cognitive tasks, walking or climbing stairs, dressing or bathing, or doing errands alone. Adjusted for age. Based on U.S. Department of Health and Human Services disability data standard (https://aspe.hhs.gov/reports/hhs-implementation-guidance-data-collection-standards-race-ethnicity-sex-primary-language-disability-0).

^§§^ Psychological distress was measured by the Kessler Psychological Distress Scale (range = 0–24) (Cronbach’s alpha = 0.85).

^¶¶^ 2019 Federal poverty level thresholds were calculated on the basis of U.S. Department of Health and Human Services Federal poverty level guidelines (https://aspe.hhs.gov/topics/poverty-economic-mobility/poverty-guidelines/prior-hhs-poverty-guidelines-federal-register-references/2019-poverty-guidelines).

*** Physically abused or harassed by a sexual partner.

^†††^ Physically forced or verbally threatened to have sex when they did not want to.

^§§§^ Physically abused or harassed because of gender identity or presentation.

^¶¶¶^ Measured by 12-item Multidimensional Scale of Perceived Social Support. Responses to the five questions were summed and averaged (range = 1–5) (Cronbach’s alpha = 0.92).

**** Incarceration was defined as having been held in a detention center, jail, or prison for >24 hours.

^††††^ Sex work was defined as receiving money or goods in exchange for sex during the past 12 months.

The odds of being homeless for a longer duration were higher for younger age groups of transgender women than the corresponding odds of longer duration of homelessness among transgender women aged ≥50 years (Table 3). The odds of longer duration of homelessness for transgender women with a disability were 1.24 times the corresponding odds among those without a disability. The odds of longer duration of homelessness for transgender women with less education were higher than the corresponding odds among transgender women having a college degree or more. Transgender women reporting an income at or below the Federal poverty level had 1.29 times the corresponding odds of experiencing longer duration of homelessness among transgender women having income above the Federal poverty level.

**TABLE 3:** Comparison of duration of homelessness among transgender women who have experienced homelessness,* by selected characteristics — National HIV Behavioral Surveillance Among Transgender Women, seven urban areas,^†^ United States, 2019–2020

Characteristic	Longer duration of homelessness^§^
	aPOR^¶^ (95% CI)
**Demographics**	
Age group, yrs	
18–24	3.11 (1.80–5.36)
25–29	2.12 (1.41–3.17)
30–39	1.97 (1.29–3.01)
40–49	1.42 (0.99–2.03)
≥50	Ref
**Health status**	
NHBS HIV test result**	
Positive	1.03 (0.97–1.10)
Negative	Ref
**Disability status^††^**	
Has a disability	1.24 (1.17–1.31)
No disability	Ref
**Mental health**	
Psychological distress^§§^	1.02 (1.02–1.03)
**Social factors**	
Education	
<High school	2.04 (1.22–3.44)
High school or equivalent	2.20 (1.40–3.44)
Some college or technical degree	1.75 (1.13–2.71)
College degree or more	Ref
**Health insurance status**	
Uninsured	1.03 (0.95–1.12)
Insured	Ref
**Poverty level^¶¶^**	
At or below Federal poverty level	1.29 (1.18–1.40)
Above Federal poverty level	Ref
**Experience of abuse past 12 months**	
Physical intimate partner violence***	
Yes	1.29 (1.22–1.36)
No	Ref
Forced sex^†††^	
Yes	1.25 (1.16–1.35)
No	Ref
Social support scale^§§§^	0.89 (0.86–0.92)
**Evicted or denied housing because they are transgender or gender nonconforming past 12 months**	
Yes	1.37 (1.29–1.46)
No	Ref
**Incarceration^¶¶¶^**	
Never incarcerated	Ref
Incarcerated, not in past 12 months	1.42 (1.05–1.91)
Incarcerated in past 12 months	5.13 (3.66–7.20)
Exchange sex for money or drugs****	
Yes	1.24 (1.18–1.30)
No	Ref

**Abbreviations:** aPOR = adjusted prevalence odds ratio; NHBS = National HIV Behavioral Surveillance; Ref = referent group.

* Adjusted prevalence odds ratios and confidence intervals from ordinal logistic regression of N = 1,566 participants with no missing information on homelessness during the past 12 months.

^†^ Atlanta, GA; Los Angeles, CA; New Orleans, LA; New York City, NY; Philadelphia, PA; San Francisco, CA; and Seattle, WA.

^§^ All models presented were separate; each was adjusted for urban area and network size and clustered on recruitment chains; longer duration of homelessness was defined as a three-level ordinal variable of 0, 1–29, and 30–365 nights homeless during the past 12 months.

^¶^ All models satisfied the proportional odds assumption score test.

** Participants with a reactive rapid NHBS HIV test result supported by a second rapid test or supplemental laboratory-based testing.

^††^ Serious difficulty hearing, seeing, doing cognitive tasks, walking or climbing stairs, dressing or bathing, or doing errands alone. Adjusted for age. Based on U.S. Department of Health and Human Services disability data standard (https://aspe.hhs.gov/reports/hhs-implementation-guidance-data-collection-standards-race-ethnicity-sex-primary-language-disability-0).

^§§^ Psychological distress was measured by the Kessler Psychological Distress Scale (range = 0–24).

^¶¶^ 2019 Federal poverty level thresholds were calculated on the basis of U.S. Department of Health and Human Services Federal poverty level guidelines. (https://aspe.hhs.gov/topics/poverty-economic-mobility/poverty-guidelines/prior-hhs-poverty-guidelines-federal-register-references/2019-poverty-guidelines).

*** Physically abused or harassed by a sexual partner.

^†††^ Physically forced or verbally threatened to have sex when they did not want to.

^§§§^ Measured by the 12-item Multidimensional Scale of Perceived Social Support. Responses to the five questions were summed and averaged (range = 1–5) (Cronbach’s alpha = 0.92).

^¶¶¶^ Incarceration was defined as having been held in a detention center, jail, or prison for >24 hours.

**** Sex work was defined as receiving money or goods in exchange for sex during the past 12 months.

Transgender women who experienced certain types of abuse had higher odds of longer duration of homelessness than transgender women who did not experience abuse (Table 3). Social support was negatively associated with longer duration of homelessness among transgender women. Transgender women who were evicted or denied housing because they are transgender or gender nonconforming during the past 12 months had 1.37 times the odds of longer duration of homelessness compared with those who were not evicted or denied housing because they were transgender or gender nonconforming. Ever being incarcerated, whether before the past 12 months or during the past 12 months, was associated with longer duration of homelessness than for transgender women who had never been incarcerated. The odds of longer duration of homelessness among transgender women who exchanged sex for money or drugs during the past 12 months were 1.24 times the corresponding odds among those who did not exchange sex.

## Discussion

During 2019–2020, transgender women participating in NHBS-Trans reported high prevalence of homelessness. Numerous personal characteristics and social factors were associated with longer duration of homelessness, with four out of 10 transgender women experiencing homelessness during the past 12 months (*4*), approximately three out of 10 experiencing 30–365 nights homeless, and approximately one out of 10 experiencing 1–29 nights homeless. Longer duration of homelessness was positively associated with younger age groups, lower educational attainment, income at or below the Federal poverty level, having a disability, experiences of abuse during the past 12 months, incarceration, eviction or denial of housing because they are transgender or gender nonconforming, and exchange sex. Longer duration of homelessness was negatively associated with social support. Efforts to prevent HIV transmission and to address housing instability for transgender women are urgently needed. These efforts should focus on systemic problems of economic instability, housing discrimination, and antitransgender discrimination that affect transgender women’s ability to access safe and affordable housing.

The proportion of younger transgender women experiencing homelessness, especially those aged <40 years, is of concern and is consistent with previous studies (*21*,*22*). Younger transgender women might experience a lack of familial support (*23*,*24*) and economic marginalization because of fewer employment opportunities and employment discrimination (*21*). Transgender youths experience higher rates of violence victimization, substance use, suicide risk, and sexual risk than their cisgender counterparts (*25*), which affect options for housing and employment. Transgender youths are also more likely to engage in survival sex, which is associated with homelessness (*26*).

Psychological distress was associated with longer duration of homelessness among transgender women. Multiple studies, including a systematic review that applied the minority stress model as a framework for reviewing 77 studies of mental health conditions among transgender or gender nonconforming persons (*27*), found mental health conditions and psychological distress to be higher among transgender women than among their heterosexual counterparts (*28*). Other researchers have found psychological distress, as identified in this study, to be associated with experiences of housing instability (*27*).

Social factors (e.g., low educational attainment) were associated with longer duration of homelessness, supporting the findings of a study that reported that young transgender women who had dropped out of school because of stigma or harassment for being transgender were more likely to experience negative consequences, including incarceration (*29*). Higher educational attainment directly affects employment opportunities and poverty status. Requiring training for teachers and administrators that focuses on strategies to reduce stigma, discrimination, and bullying in school systems could improve retention in school for young transgender women (*25*).

Social support was lower among transgender women experiencing longer duration of homelessness than among those not experiencing homelessness. A lack of social support for transgender women can increase depression and anxiety (*30*) and affect resilience (*31*), which are associated with housing instability. Further, social isolation can affect engagement and retention in HIV care and viral suppression for transgender women with diagnosed HIV infection (*32*). Family members, friends, health providers, and community members can access resources to self-educate and learn how to express support for their transgender loved ones (e.g., through resources for parents from the Trans Youth Equality Foundation [http://www.transyouthequality.org/for-parents] and PFLAG [https://pflag.org/glossary_term/transgender]).

Experiences of violence and abuse also were associated with longer duration of homelessness, indicating another layer of harm that can interfere with access to basic needs for transgender women. In this analysis, transgender women experiencing various forms of abuse, either physical intimate partner violence or forced sex, experienced longer duration of homelessness. More than half of transgender women who experienced any form of abuse during the past 12 months reported experiencing homelessness. These findings are supported by an analysis that reported rates of lifetime homelessness in the 2015 U.S. Transgender Survey were associated with all forms of interpersonal violence, including physical and psychological violence, and experiencing forced sex during the past 12 months (*33*). Certain transgender women might experience housing instability because of abusive partners (*34*). Transgender women who have experienced intimate partner violence might be deterred from seeking or have difficulty accessing intimate partner violence services because of transphobic discrimination or rigidly gender-segregated services (*34*). Further, transgender women often experience physical or sexual violence in homeless shelters; unsafe shelters can force them out on the street (*35*).

Another social determinant, housing discrimination, was prevalent in the sample; transgender women who were evicted or denied housing because they are transgender or gender-nonconforming had higher odds of experiencing longer duration of homelessness. These results are consistent with previous findings (*9*,*26*) illustrating that systemic factors driving housing instability (e.g., economic insecurity, housing discrimination, and antitransgender discrimination) are known barriers to housing for transgender women (*36*). The Fair Housing Act prohibits discrimination on the basis of gender identity (https://www.hud.gov/program_offices/fair_housing_equal_opp/fair_housing_act_overview); transgender women who have experienced housing discrimination can file a complaint with the U.S. Department of Housing and Urban Development (https://www.hud.gov/program_offices/fair_housing_equal_opp/online-complaint). Further, homeless shelters and domestic violence shelters and services can consider expanding services for transgender clients, ensure shelters and services are safe, and provide cultural competency training to staff members to better support transgender clients.

Incarceration was associated with longer duration of homelessness among the transgender women participating in this study. Incarceration among transgender women has been found to affect mental health (e.g., anxiety and depression) and substance use, and has been associated with homelessness, sex work, school dropout, and multiple incarcerations (*29*). Transgender women who are incarcerated experience victimization, harassment, and violence at very high rates (*37*). They are often misgendered, denied health care, punished for expressing their gender identity, and susceptible to sexual violence (https://www.aclu.org/news/lgbtq-rights/sex-work-is-real-work-and-its-time-to-treat-it-that-way). Additionally, the majority of NHBS-Trans participants were Black or Hispanic transgender women, who are targeted by law enforcement and incarcerated at high rates (*38*). Law enforcement policies and priorities that criminalize homelessness make transgender women experiencing homelessness, especially transgender women of color, vulnerable to harassment, policing, and incarceration (*38*). Addressing violence toward transgender women through education and training, public awareness, and policies that criminalize discrimination of transgender persons in schools, the workplace, and housing can positively affect housing stability and quality of life for transgender women.

Approximately half of transgender women who had exchanged sex for money or drugs had experienced 30–365 nights homeless. Discrimination and stigma in the workplace are often barriers to employment for transgender women, limiting options for income and encouraging engagement in sex work (*39*). Because of the illegality of sex work in the United States, sex work and incarceration are highly correlated, and both are associated with mental health conditions and sexual behaviors associated with HIV transmission (*40*). A cyclical relation between sex work and housing instability can exist if transgender women engage in sex work to generate income for housing. This type of survival sex for income interferes with housing stability and can affect mental and physical health outcomes and can increase chances of acquiring HIV infection (*26*,*41*). Another study found that transgender women who participated in sex work experienced lower social support and higher rates of violence, stigma, and HIV than their non–sex-working peers (*42*,*43*). Decriminalizing sex work and decreasing stigma and victimization could reduce criminal justice involvement among transgender women and facilitate employment and housing opportunities.

The findings in this study demonstrated that multiple personal characteristics and social factors are associated with longer duration of homelessness; providing stable housing for transgender women could improve physical and mental health outcomes and safety. Specific housing interventions could address different durations of homelessness, either short-term or episodic or longer-term or chronic homelessness. Transgender women who experience less than 30 days homeless could benefit from emergency assistance programs that provide support and services for rent or utilities to prevent eviction, the need to move frequently, couch surfing, and other circumstances that could lead to longer-term homelessness. Despite being illegal, stigma and discrimination in housing and employment based on transphobia (*11*) limit options for transgender women, decreasing opportunities for engagement in the licit economy. Stable housing, using the Housing First model that prioritizes safe and affordable housing with wrap-around social services for mental health and substance use, can improve quality of life and HIV outcomes (*44*). Approaches to treatment using a trauma-informed care model (https://store.samhsa.gov/sites/default/files/d7/priv/sma14-4884.pdf) specifically designed for transgender women can also be implemented to address a history of abuse and violence often experienced by transgender women. Structural interventions that address HIV prevention among transgender women need to focus on stigma, discrimination, and poverty (*45*). Forty-two percent of the participants in NHBS-Trans were HIV-positive and might qualify for the Housing Opportunities for Persons with AIDS (HOPWA) program (https://www.hud.gov/program_offices/comm_planning/hopwa). CDC funded a toolkit, developed by community partners, for providing HIV Prevention Services to Transgender Women of Color (https://www.cdc.gov/hiv/effective-interventions/prevent/toolkit-transgender-women-of-color/index.html). This toolkit, for use by community organizations, health departments, clinics, and other organizations that provide services for transgender women of color, outlines services and interventions to address important topics (e.g., healthy partner relationships, sexual risk behaviors, stress, social support, gender affirmation, HIV or STI knowledge, and engagement in care). Employing these varied evidence-based interventions could address certain social factors and personal characteristics affecting housing stability among transgender women.

## Limitations

General limitations for the NHBS-Trans are available in the overview and methodology report of this supplement (*16*). The findings in this report are subject to at least five additional limitations. First, the sample is not representative of transgender women residing outside of the seven urban areas. Because of the hard-to-reach nature of transgender women, the data might not be representative of all transgender women residing in the seven urban areas. Second, the data are self-reported and certain measures, such as psychological distress, exchange sex, or experience of abuse, might be subject to social desirability biases resulting in underestimates of these factors (*46*,*47*). Third, the sample size of transgender women experiencing 1–29 days homeless was limited, thus, inferences from this group to all transgender women cannot be made. Fourth, the question assessing homelessness is limited and does not include transitory instances of housing instability (e.g., couch surfing) or information on participants experiencing homelessness for longer than 12 months. To address this limitation, data on homelessness were stratified by duration of homelessness to identify differences possibly related to transitional or episodic homelessness (*14*). Finally, the cross-sectional study design limits the ability to establish causality and prohibits analysis of the dynamic nature of housing instability.

## Conclusions

Transgender women experience housing instability associated with personal characteristics and social factors; these factors increase behavioral risk factors for HIV infection and poor health outcomes. Specifically, social factors (e.g., living below the Federal poverty level, experiences with violence and abuse, and lack of social support) were associated with longer duration of housing instability and homelessness. Further, history of incarceration, exchanging sex for money or drugs, experience with being rejected from housing, or being evicted were all factors associated with housing instability and homelessness. These social factors are entwined in societal views of discrimination and stigma of transgender women. Interventions that address personal characteristics and social factors and promote positive attitudes toward transgender women can help to achieve housing stability and can improve mental and physical health and HIV outcomes for transgender women. Further, integrating housing services, behavioral health services, employment, gender-affirming medical care, and clinical care are important to improve the living circumstances and quality of life for transgender women.

## Footnotes

* 45 C.F.R. part 46, 21 C.F.R. part 56; 42 U.S.C. Sect. 241(d); 5 U.S.C. Sect. 552a; 44 U.S.C. Sect. 3501 et seq.
